# Synthetic Symbiosis under Environmental Disturbances

**DOI:** 10.1128/mSystems.00187-20

**Published:** 2020-06-16

**Authors:** Jai A. Denton, Chaitanya S. Gokhale

**Affiliations:** a Genomics & Regulatory Systems Unit, Okinawa Institute of Science & Technology, Onna-son, Japan; b Research Group for Theoretical Models of Eco-evolutionary Dynamics, Department of Evolutionary Theory, Max Planck Institute for Evolutionary Biology, Plön, Germany; MIT

**Keywords:** nonlinear interactions, environmental change, mathematical modeling, symbiosis, synthetic biology

## Abstract

By virtue of complex ecologies, the behavior of mutualisms is challenging to study and nearly impossible to predict. However, laboratory engineered mutualistic systems facilitate a better understanding of their bare essentials. On the basis of an abstract theoretical model and a modifiable experimental yeast system, we explore the environmental limits of self-organized cooperation based on the production and use of specific metabolites. We develop and test the assumptions and stability of the theoretical model by leveraging the simplicity of an artificial yeast system as a simple model of mutualism. We examine how one-off, recurring, and permanent changes to an ecological niche affect a cooperative interaction and change the population composition of an engineered mutualistic system. Moreover, we explore how the cellular burden of cooperating influences the stability of mutualism and how environmental changes shape this stability. Our results highlight the fragility of mutualisms and suggest interventions, including those that rely on the use of synthetic biology.

**IMPORTANCE** The power of synthetic biology is immense. Will it, however, be able to withstand the environmental pressures once released in the wild. As new technologies aim to do precisely the same, we use a much simpler model to test mathematically the effect of a changing environment on a synthetic biological system. We assume that the system is successful if it maintains proportions close to what we observe in the laboratory. Extreme deviations from the expected equilibrium are possible as the environment changes. Our study provides the conditions and the designer specifications which may need to be incorporated in the synthetic systems if we want such “ecoblocs” to survive in the wild.

## Abstract (precis)

The power of synthetic biology is immense. Will it, however, be able to withstand the environmental pressures once released in the wild. As new technologies aim to do precisely the same, we use a much simpler model to test mathematically the effect of a changing environment on a synthetic biological system. We assume that the system is successful if it maintains proportions close to what we observe in the laboratory. Extreme deviations from the expected equilibrium are possible as the environment changes. Our study provides the conditions and the designer specifications which may need to be incorporated in the synthetic systems if we want such “ecoblocs” to survive in the wild.

## INTRODUCTION

Life on earth comprises a hierarchy of units of selection. From societies to genes, we find recursive patterns of organization at each of these levels (1). However, selection is not limited to a specific level. While changes may occur at lower levels, such as a single nucleotide or amino acid, selection could operate at the level of the organism or above. Competition between entities at a specific level of organization can spell disaster for a higher level. A clear example of this breakdown of control is cancer (1).

Therefore, mutualistic interactions, a specific type of cooperation where replicating components benefit each other, can be targeted by selection at a higher level (2). Due to conflicts of interest between entities at various levels of selection, the origin of mutualism and subsequent selection lack a clear evolutionary explanation—an entire field of research in itself (3). Even though it is hard to explain how mutualisms emerge, they are highly prominent and form the basis of countless ecosystems. On a global scale, examples such as coral-*Symbiodinium* symbioses or plant-rhizobium interactions are well-known (4–6). Many such mutualisms have evolved over millions of years. But if mutualisms are fragile and susceptible to collapse, as hypothesized, then how do they survive for eons in continually changing environments?

Although mutualistic interactions emerge in numerous ways (3), we focus on how they survive. Regardless of their origin, mutualisms are regularly threatened. A common challenge for mutualistic communities is exploitation by cheater strains that benefit from the interactions but fail to contribute. The problem of parasitic elements, first noted by Maynard Smith (7), has been extensively studied. Postulates about compensatory mechanisms that could avoid parasitic exploitation range from conceptual (8) to mechanistic (9). Besides the evolutionary conundrums about mutualism, we propose that mutualism could also suffer from insufficient ecological support. That is, if population densities of mutualistic partners are inadequate, then mutualisms are hard to sustain. This problem highlights the necessity of a physical structure akin to a “warm little pond” for concentrating the initial mutualists (10). Without a physical structure or substrate to provide overlapping niches, it would be challenging to kick-start necessary mutualistic reactions (11). However, what happens to the stability of a mutualistic system when the threat to mutualism is an unsupportive environment? Understanding how cooperative interactions survive in changing environments that continually alter the basis of mutualistic interactions is worthy of investigation.

The selective advantage derived from cooperation may be transitory in the face of environmental changes; therefore, in most cases, the dynamics of such systems remain in a constant state of flux. Interspecific interactions are essential in determining community stability (12). Resource availability can modulate the nature of ecological interactions: from facultative mutualism to competition, and even parasitism (13). In a synthetic system, interactions are fixed; therefore, we can focus on the environmental dynamics. However, ecological networks are inherently complex (14). While a complete understanding of a network provides us with general properties of the interactions, it is often impossible to determine the principles that form the building blocks. As such, it is easier to study tightly controlled systems with a defined number of interactions. Numerous systems adapted from nature allow us to study evolutionary and population dynamics.

The intrinsic complexity of wild and even laboratory-adapted organisms makes them unwieldy to directly test the assumptions of a mathematical theory. Via genetic manipulation, synthetic, cross-feeding, cooperation can be engineered within microbial communities (13, 15–18). Complex population dynamics in response to temporal, spatial, and environmental factors can be dissected by fine-tuning and manipulating these synthetic systems (18, 19). Numerous synthetic systems developed are especially mutualistic in their interaction pattern (20). We use Saccharomyces cerevisiae synthetic mutualistic systems that rely on cross-feeding of metabolites between two strains (15) as a simple model to reflect the mathematical theory developed in the following sections.

The designed system uses feedback resistance (fbr) mutations in adenine and lysine biosynthesis pathways. The fbr mutations result in overproduction of the corresponding metabolite. The strains used are referred to here as *LYS*↑ and *ADE*↑. The dynamics of this cross-feeding system are comparable to a simple but powerful theoretical model of self-organization—a hypercycle. This model has extensive applications from explaining how life may have originated to how complex communities could form (11, 18, 21–23). On the basis of this theoretical background, we develop a simple but powerful and easily extendable phenomenological model. We then use the S. cerevisiae yeast system to validate the model and finally explore beyond the synthetic system to understand the stability and cost of cooperative interactions in changing environments. We establish the baseline mutualistic properties of this system and then identify conditions that can potentially disrupt this natural state. The proportions of the mutualists are our property of interest, which favorably comes close to ≈50% here. We define a proportion range around the equilibrium, here 20% to 80% where both the mutualists can be observed at similar frequencies. Where the mutualists exist naturally at a different equilibrium, the proportion range would need to be defined accordingly. Ecology of an organism is made up of both biotic as well as abiotic factors. While it is clear that changes in biotic ecology (population densities) affect the interaction pattern, it is essential to take into account the effects of the abiotic part of the ecology (24, 25). With this aim in mind, we begin with our theoretical and experimental model.

## RESULTS

Our model begins with the growth of two yeast strains in an environment that lacks free adenine and lysine as nutrient sources. We have devised a model of intermediate complexity. We are extending beyond the extremely simplistic pairwise interaction model by accounting for the metabolite dynamics but limiting the parameterization. The model seeks to strike a balance between the simple pairwise systems and complex highly parameterized systems. The two strains are *LYS*↑ and *ADE*↑, and the densities of the *LYS*↑ and *ADE*↑ strains are denoted by *x_L_* and *x_A_*, respectively. The *LYS*↑ strain is deficient in adenine, while it overproduces lysine and vice versa for the *ADE*↑ strain. Modifying the logistic growth equation gives the dynamical equations for the growth of the two strains:

(1) $$\begin{array}{l} {\dot{x}}_{L}=x_{L}\left(r_{1}\frac{c_{A}}{c_{A}+k_{c_{A}}}\right)\left(1-\frac{x_{L}+x_{A}}{K}\right)\\ {\dot{x}}_{A}=x_{A}\left(r_{2}\frac{c_{L}}{c_{L}+k_{c_{L}}}\right)\left(1-\frac{x_{L}+x_{A}}{K}\right) \end{array}$$

The two strains grow if the required metabolites are present, which result in growth at a rate of *r_i_*. The densities of the available metabolites are given by *c_A_* (adenine) and *c_L_* (lysine). The strains compete for a limited amount of space, given by *K*. Similar to the study reported in reference 18, we neglect the death rates assuming that the birth rates sufficiently characterize the growth. Additionally, we show that death is negligible over the time course that we focus on (see Materials and Methods). Metabolite concentrations, together with Monod-type saturation kinetics, control the growth of the strains. Although deriving a mathematical model for such a pairwise system can exclude metabolites, the explicit inclusion of metabolites is crucial, as pairwise Lotka-Volterra models may not always provide a realistic qualitative picture of the dynamics (14). Metabolites, in this particular case, are a consumable resource. Furthermore, the level of abstraction provided in equation 1 is adequate to focus on the relative concentrations of the mutualists. In reference 26, the authors focus on the postlag steady-state growth rate. A detailed predictive mathematical model for growth rate would need to match with precise measurements of the birth and death rates and the metabolite release and consumption rates. Our goal is more straightforward, and hence, in this case, we forgo the use of a complicated system with multiple parameters.

**FIG 1:**
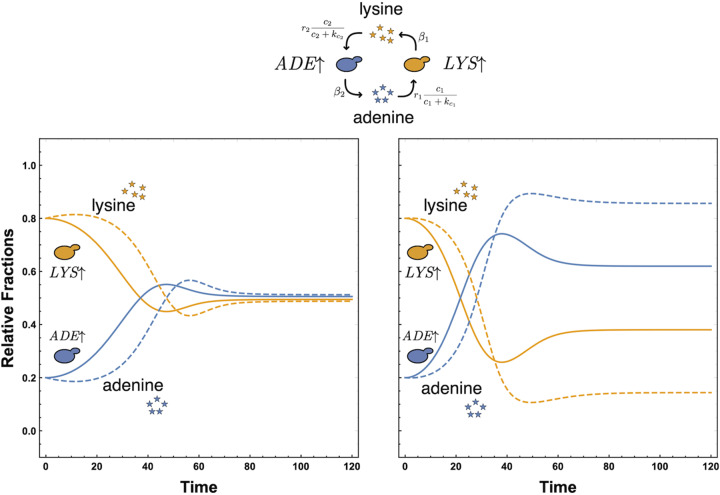
Theoretical model dynamics for the neutral expectation (left) and experimentally informed expectation (right). We plot the fractions of the *LYS*↑ strain [*x_L_*/(*x_L_* + *x_A_*)] and *ADE*↑ strain [*x_A_*/(*x_L_* + *x_A_*)] (solid lines) and the relative fractions of metabolites adenine [*c_A_*/(*c_A_* + *c_L_*)] and lysine [*c_L_*/(*c_A_* + *c_L_*)] (dashed lines) in a continuous culture. As an initial condition, there is no free adenine or lysine in the culture, but nonzero populations of the strains generate the free adenine and lysine in the same relative frequencies. The relative initial fractions for the strains are 0.8 for the *LYS*↑ strain and 0.2 for the *ADE*↑ strain, and the rate at which the two strains share the two metabolites is set to β_1_ = β_2_ = 0.1. We set the carrying capacity of the culture vessel to *K *= 1. (Left) The conversion rate of the metabolites to growth and the rate at which the metabolites are consumed are exactly the same (*r*_1_ = *r*_2_ = 1 and γ_1_ = γ_2_ = 1). The Michaelis constant for both metabolites to $k_{c_{A}}=k_{c_{L}}=1$. Under these symmetric conditions, the relative fractions of the strains, as well as those of the two metabolites, reach an equilibrium where normalized concentrations are the same. (Right) Clearly, the symmetry assumption in all the rates is a simplification. Informed by experiments, we estimate that the growth constant of the *ADE*↑ strain to be twice as much as of the *LYS*↑ strain, *r*_1_ = 1, *r*_2_ = 2, and the Michaelis constants $k_{c_{A}}=2$ and $k_{c_{L}}=1$. This asymmetry in uptake rates is reflected in the resulting unequal equilibrium.

The densities of the metabolites in the culture are linked to the dynamics of the strains. As the metabolites are produced constitutively by one of the strains (at rate β*_i_*), the other strain uses them immediately (at a maximum rate of γ*_i_*). The metabolite density dynamics hence can be captured by:

(2) $$\begin{array}{l} {\dot{c}}_{A}={\text{β}}_{1}x_{A}-\frac{{\text{γ}}_{1}c_{A}}{c_{A}+k_{c_{A}}}x_{L}\\ {\dot{c}}_{L}={\text{β}}_{2}x_{L}-\frac{{\text{γ}}_{2}c_{L}}{c_{L}+k_{c_{L}}}x_{A} \end{array}$$

where $k_{c_{A}}$ and $k_{c_{L}}$ are the normalized half-saturation constants for adenine and lysine, respectively. We assume that use of a metabolite at rate γ*_i_* by strains *x_i_* also involves the formation of an intermediate, thus being subject to Michaelis-Menten kinetic parameters. The simple dynamics of such a system are depicted in Fig. 1.

The model developed already possesses intermediate complexity. Typically for gaining qualitative insight, and mathematical tractability, even simpler models can be developed. In Text S1, Fig. S1, and Fig. S2 in the supplemental material, we show the derivation of a minimal ecoevolutionary mathematical model which affords mathematical tractability but does not capture the metabolites explicitly. We capture the ecological (population) dynamics, but information about the evolutionary dynamics (relative fractions of the strains) is missing, lacking feedback between the metabolites and strain growth.

10.1128/mSystems.00187-20.1 TEXT S1 Minimal model. Derivation and description of an extremely minimal model of the mutualistic system highlighting the precise loss of ecological information. Download Text S1, PDF file, 0.1 MB. Copyright © 2020 Denton and Gokhale. 2020 Denton and Gokhale This content is distributed under the terms of the Creative Commons Attribution 4.0 International license.

10.1128/mSystems.00187-20.2 FIG S1 Ecoevolutionary dynamics. In the space of population density and the relative fraction of the *ADE*↑ strain, we show the ecoevolutionary dynamics under different death rates. For low death rates, the population is at carrying capacity with the equilibrium defined by the growth rates *r*_1_ and *r*_2_ (here *r*_1_ = 1 and *r*_2_ = 2, reflecting the growth rates as in the main text). The equilibria of the system are defined by the intersection of *z* = 0 and *f* = 0. As the death rate increases, the solution for *z* = 0 (dashed solution) shrinks and ultimately vanishes. Since *f* is independent of *d*, the other solution (blue solid line) remains. Download FIG S1, PDF file, 0.7 MB. Copyright © 2020 Denton and Gokhale. 2020 Denton and Gokhale This content is distributed under the terms of the Creative Commons Attribution 4.0 International license.

10.1128/mSystems.00187-20.3 FIG S2 Phase portrait for death. For *r*_1_ = 1, we explore different values of *r*_2_ from 1.0, 1.2, 1.4, 1.6, 1.8, and 2.0. Increasing from very small death rates, we observe a set of two solutions, unstable (dashed lines) and stable (full lines). With increasing death rates, the stable solution reduces in the equilibrium population density up to 0.,5 where the two solutions meet and annihilate each other. The only stable solution then is population extinction. Download FIG S2, PDF file, 0.4 MB. Copyright © 2020 Denton and Gokhale. 2020 Denton and Gokhale This content is distributed under the terms of the Creative Commons Attribution 4.0 International license.

**FIG 2:**
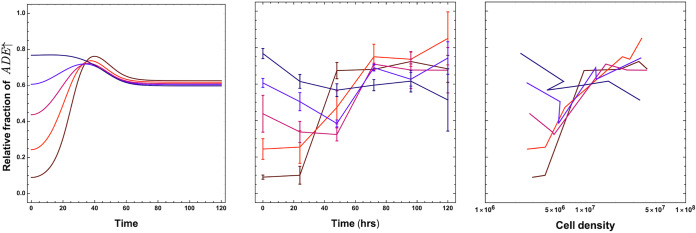
Theoretical and experimental model dynamics with different initial *ADE*↑ concentrations. Using the theoretical model, in the left panel, we predict the dynamics of the normalized fraction of the *ADE*↑ strain starting at various initial ratios with the parameter set informed by data. The initial concentrations of the metabolites *c_A_* and *c_L_* are set at 0.0. Using the experimental model, in the middle panel, we plot the fraction of the yeast *ADE*↑ strain relative to the *LYS*↑ strain. Starting at different initial ratios, we track dynamics over a 120-h period in synthetic complete medium (without adenine or lysine). Each point is the mean for three biological replicates, and error bars represent a single standard deviation. In the right panel, we plot the fraction of the *ADE*↑ strain against the total cell density of the culture. This highlights the ecological dynamics of the study system. Irrespective of starting conditions in the relative fraction space, the general trend for cell density is to increase. Thus, we focus on the single dimension of the fraction of the *ADE*↑ strain.

The model as per equations 1 and 2 has the potential to be parameterized for any particular system, but we prefer to keep the results general. Therefore, the model has not been fitted to the growth rates as estimated from experiments but informed by them to capture the qualitative dynamics. By adding the required supplement, adenine or lysine, we calculate the growth rate of the two strains for different levels of supplementation. The experimental *ADE*↑ and *LYS*↑ growth rates for different amounts of supplementation are shown in Materials and Methods, and the raw data have been provided. Using changes in the growth rates, we estimate the relative values of *r_i_* and *k_i_* for the two strains. The production rate of the metabolites is assumed to an order of magnitude smaller than the growth rates, and the maximum rate of uptake is set to unity (γ*_i_* = 1). The parameters used are *r*_1_ = 1 and *r*_2_ = 2 and the Michaelis constants $k_{c_{A}}=2$ and $k_{c_{L}}=1$. The metabolites are produced by the strains at β*_i_* = 0.1. We assume the Michaelis constant estimated for the uptake rate for adenine and lysine to be the same as the rate at which they degrade from the pool (γ*_i_* = 1). At various initial conditions of the *ADE*↑-to-*LYS*↑ ratio, the final equilibrium values are consistently close to 0.6, corroborated by experiments shown in Fig. 2.

### Ecoevolutionary dynamics under environmental disturbance.

The mutualistic system rests on the interdependence of the two strains of yeast. If we undermine this dependency, it will affect the stability of the mutualistic interaction. One way to undermine the system is to introduce a parasite (18). By doing so, we change the environment itself without altering the genotypes of the interacting strains.

If the environment has one of the two required metabolites, then the strain using that metabolite will not depend on the corresponding strain for survival. A significant amount of supplementation can drown out the signal of the other strain. The addition of supplementation dilutes the growth medium, but below an experimentally detected level, we do not consider any detrimental effects of supplementation in the model. In all, we visualize three different supplementation scenarios that could be used to test the resilience of this mutualistic system: (i) initial supplementation (adenine or lysine added at the beginning of culturing), (ii) continuous supplementation (both metabolites added steadily throughout culturing), and (iii) intermittent supplementation (the metabolites added at regular time intervals). Depending on the time scale, a single niche can experience all three scenarios, but we analyze the three scenarios in succession.

### Initial supplementation. (i) Theory.

To supplement at various concentrations of adenine or lysine, we only need to change the initial conditions of the metabolites in equations 2.

### (ii) Experiments.

**FIG 3:**
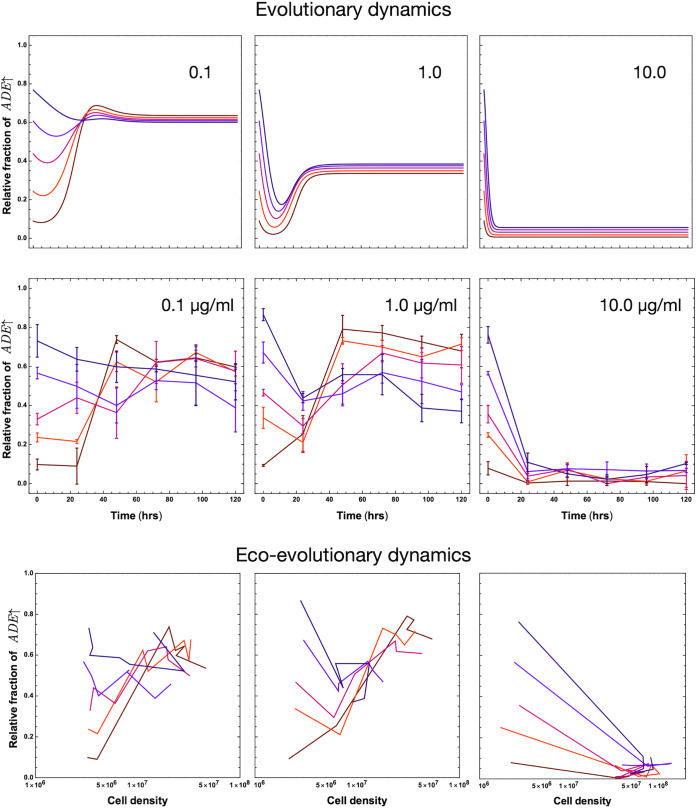
Theoretical and experimental model dynamics with different initial supplementation levels of adenine. We explore the theoretical model as depicted in Fig. 2, now with added initial supplementation. The initial concentrations of the metabolites *c_A_* and *c_L_* are set at 0.1, 1.0, and 10 as denoted in the panels. The model results in a decrease in the equilibrium fraction of the amount of the *ADE*↑ strain with increasing initial supplementation. The experimental result (middle row) shows the fraction of the yeast *ADE*↑ strain relative to the *LYS*↑ strain, at various starting ratios over a 120-h period in synthetic complete medium supplemented with adenine to a final concentration of either 0.1, 1.0, or 10 μg/ml. Each point is the mean for three biological replicates, and the error bars represent a single standard deviation. The theoretical model qualitatively predicts experimental model dynamics of mutualism. With increasing supplementation, the *LYS*↑ strain dominates the system as its dependence on the *ADE*↑ strain is reduced. Again, the ecoevolutionary dynamics are shown in the bottom row where the effect of initial supplementation is localized in evolutionary space rather than ecological space.

The initial supplementation regime is a standard batch culture. With required metabolites provided at the start, the culture continues to grow until one or more metabolites become limiting. We supplemented our experimental cultures with either 0.1, 1, or 10 μg/ml adenine and measured the effect on strain ratio at regular time intervals (Materials and Methods and Fig. 3 [top]). Supplementation favored the *LYS*↑ strain, resulting in a lower prevalence of the *ADE*↑ strain compared to the unsupplemented condition. The mean *ADE*↑-to-*LYS*↑ strain ratios after 120-h growth of all starting ratios for 0.1, 1, or 10 μg/ml adenine supplementation are 0.53, 0.57, and 0.056, respectively. For both Fig. 2 and Fig. 3, we include ecoevolutionary dynamics. The effect seen is localized to evolutionary space rather than to ecological space, since the growth rates are always positive, but only relative growth rates are relevant. In the standard batch culture regime, the added metabolites will be used up, and then the culture will be dominated by the internal production levels of adenine and lysine. Without the addition of extra nutrients (or efflux of waste) as in a chemostat, the equilibrium of unsupplemented case is not recovered. These stringent ecological conditions also make the experimental conditions compared to the mathematical model.

**FIG 4:**
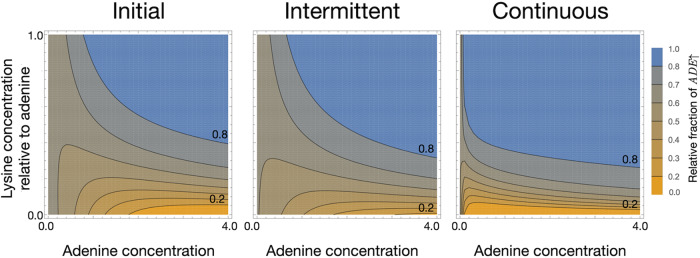
Equilibrium value of the *ADE*↑ strain for different supplementation regimes. The initial normalized concentration of the *ADE*↑ strain was 0.5. The system is assumed to have equilibrated when values of the strain densities do not change more than 10^−4^ between two consecutive numerical time steps. By 300 time steps, all initial conditions for initial supplementation have already reached this equilibrium state, and hence, we choose to show the snapshot of the system at time point 300. In the plots, we show the eventual normalized concentration of the *ADE*↑ strain going from all *ADE*↑ strain (relative fraction value of 1, blue) to all *LYS*↑ strain (relative fraction value of 0, yellow). In each plot, the initial concentration of adenine in the system at time zero is shown on the *x* axis, and the amount of lysine relative to adenine is depicted on the *y* axis. For *y *= 1, the amounts of adenine and lysine are the same. In initial supplementation, metabolites were provided only at time point *t *= 0. In intermittent supplementation, metabolites were provided starting at *t *= 25 and then every 25th time step. For continuous supplementation, metabolites were provided at every time step. Going from initial to continuous supplementation, we see that the zone (between contours 0.2 and 0.8) shrinks as the zone for the *LYS*↑ strain (yellow) increases, and so does the *ADE*↑ strain (blue) region.

Using our model, informed by experiments, we explore the relative density of the *ADE*↑ strain at a given time point as we change the amount of initially supplied metabolites. For the unsupplemented case, we know the equilibrium density both from experiments and theory (Fig. 2). For the initial supplementation regime, we assume the system to have equilibrated once the values of the strain densities do not change more than 10^−4^ between two consecutive numerical time steps. The equilibration takes typically 30 time steps, but we show the snapshot of the relative densities from time point 300 in Fig. 4 (left panel). Experimentally we tested the addition of only adenine for different initial fractions of the *ADE*↑ strain, but theoretically, we explore the consequence of adding both adenine and lysine for the *ADE*↑ strain starting at 0.5. The results, summarized in Fig. 4 (left panel), reveal that even a slight asymmetric increase in the amount of initial metabolite present in the environment is enough to destabilize the equilibrium. When the equilibrium values come close to one of the two edges of the system, one mutualist is overrepresented compared to the equilibrium. We define this overrepresentation, compared to the equilibrium, as being dominant in the population. For our system, since the equilibrium is close to equality, we symmetrically choose 0.2 as the minimum acceptable threshold fraction for mutualism to be fair in terms of the mutualist densities. As the effects of environmental perturbation are observed in evolutionary space, rather than ecological space, we use frequencies of the mutualists to define how far the system moved from the unsupplemented equilibrium. The asymmetry of the graph in Fig. 4 reflects the inherently different uptake rates of metabolites by the two strains in Fig. 2.

When supplementation is intermittent or continuous, the time to reach equilibrium is not a relevant quantity since constant perturbations will keep the system out of equilibrium. Hence, in Fig. 4 we show the state of the system at time point 300 to allow for a fair comparison between the different supplementation regimes. We are precisely interested in the far-from-equilibrium states of the perturbed systems as ecologically, these differences from the unsupplemented equilibrium will be the signals of a disturbed ecosystem. Even with supplementation, our experimental cultures will eventually reach carrying capacity, the culture will enter the death phase, and the equilibrium will shift. As such, to keep the experimental approach comparable to the theory, the cultures are limited to 120 h.

### Intermittent supplementation. (i) Theory.

For intermittent supplementation, we modify the equations 2 to

(3) $$\begin{array}{l} {\dot{c}}_{A}=c_{A\text{cont}}|\Delta t+\beta_{1}x_{A}-\frac{\gamma_{1}c_{A}}{c_{A}+k_{c_{A}}}x_{L}\\ {\dot{c}}_{L}=c_{L\text{cont}}|\Delta t+\beta_{2}x_{L}-\frac{\gamma_{2}c_{L}}{c_{L}+k_{c_{L}}}x_{A} \end{array}$$

where the values of *c_A_*_cont_ and *c_L_*_cont_ (where cont stands for continuous supplementation) determine the amount of metabolite added to the culture. The addition takes place at the end of the time interval determined by Δ*t* which defines the cycle length. This changes the model from a smooth dynamical system to a hybrid dynamical system (27). We start with the same initial conditions as for other supplementation regimes, where the initial condition of the metabolite matches the amount used for supplementation. Dynamics proceed as per equations in the main text. In total, they run for the same amount of time as the initial and continuous supplementation experiments before assuming equilibrium, but the time is split into numerous small cycles of length Δ*t*. Each cycle runs for a short period. Thus, we have cycle length × number of cycles = total time until equilibrium. At the end of each cycle, we add the predetermined amount of essential metabolites and then allow the next cycle to continue.

**(ii) Experiments.** As the initially added supplementation is consumed, it is possible to add metabolites at fixed intervals. The model is now an example of a hybrid dynamic system (27) where concentrations of metabolites are adjusted at regular intervals (see Materials and Methods and reference 4). In contrast to initial supplementation, equilibrium, in this case, does not only shift but is also maintained. In a chemostat or a continuously fed batch culture, without perturbations, we would expect the system to bounce back to the unsupplemented equilibrium. However, as here, with an intermittently disturbed environment, we can maintain the system away from the naive equilibrium.

**FIG 5:**
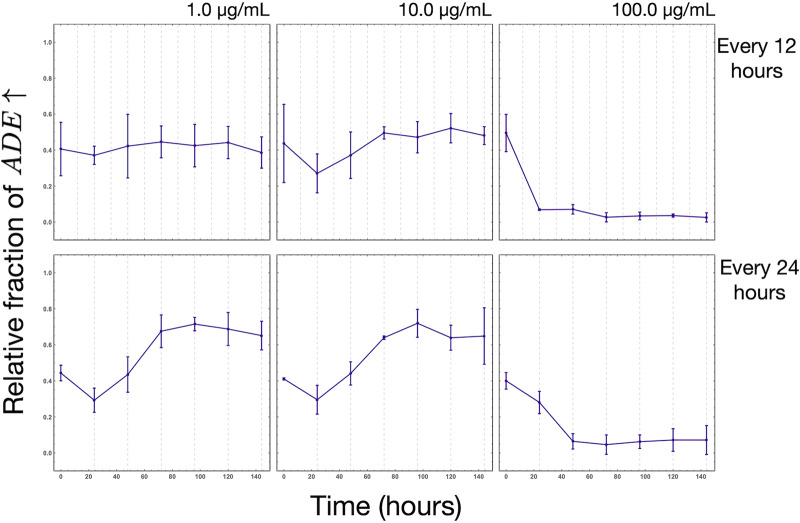
Experimental model dynamics with intermittent supplementation of adenine. This figure shows the relative fraction of the *ADE*↑ yeast strain relative to the *LYS*↑ strain over a 140-h period in synthetic complete medium. Here, we supplemented the medium with either 1.0, 10, or 100 μg/ml every 12 h (top) or 24 (bottom) h. Each point is the mean for three biological replicates, and the error bars represent a single standard deviation.

In Fig. 5, we show the experimental results of the fraction of the *ADE*↑ strain when supplemented with 1.0, 10, and 100 μg/ml of adenine every 12 or 24 h. Compared to initial supplementation (Fig. 3), the dynamics of intermittent supplementation result in a different equilibrium. For example, for 10 μg/ml under initial supplementation (Fig. 3, right column), the population fraction of the *ADE*↑ strain is almost negligible; however, for intermittent supplementation, the unsupplemented equilibrium is maintained (Fig. 5, middle column). Delay in supplementation (12 h versus 24 h) changes the time required to attain equilibrium.

**FIG 6:**
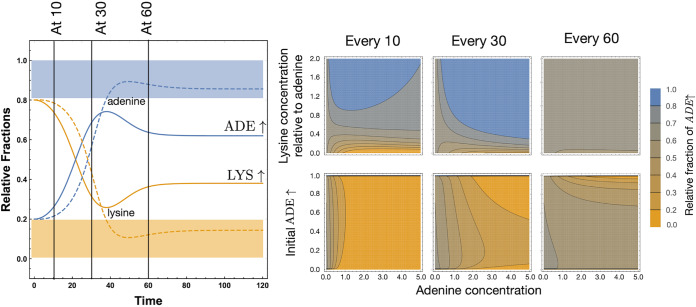
Disrupting transient mutualism with intermittent supplementation. Intermittent supplementation (Fig. 4) started with the same initial supplementation as the initial supplementation regime so as to be comparable. If we start with no metabolites in the culture and add them at fixed intervals, then it might be possible to maintain both mutualists at appreciable frequencies, even under supplementation. The exact timing of supplementation provided is crucial in determining the eventual equilibrium. If the pattern of supplementation starts early in the existence of the *ADE*↑ strain, the resulting equilibrium frequency can be drastically affected. By tuning the timing and dose of supplementation, we can maximize the probability of maintaining the system close to the unsupplemented equilibrium state (as we move from supplementing every 10 to 30 to 60 time steps). If intermittent supplementation starts in the latter phase of the transient where the equilibrium value is already reached, the coexistence seems robust. This also depends on the initial concentration of the *ADE*↑ strain (discussed further in the legend to Fig. 8). Thus, while the top row is calculated from an initial normalized concentration of 0.2 for the *ADE*↑ strain, the bottom row explores all initial conditions. Equilibrium values are all calculated at the same time point, i.e., the number of cycles are adjusted so that all integrations run until the time point when cycle length × number of cycles = 300.

Intermittent supplementation acts as a bridge between the other two supplementation regimes. If the delay between two supplementations is substantial, then starting at time *t *= 0, the scenario is the same as that of initial supplementation (Fig. 4, middle panel would resemble the left panel). If the delay between successive supplementations is minimal, then the concept is similar to continuous supplementation (Fig. 4, middle panel would resemble the right panel). In Fig. 6, we show the effect of the timing of intermittent supplementation. If the equilibrium of the system results in an intermediate fraction of both the strains, then we can maintain it if the supplementation is delayed. When supplementation occurs early in the transient stage of the dynamics, there is the potential to change the eventual outcome. For a legitimate comparison with initial and continuous supplementation, we started the intermittent supplementation with a nonzero amount and supplemented further at regular intervals.

### Continuous supplementation.

Intermittent supplementation, if done at very short time intervals, essentially represents a continuous supply of the supplemented metabolite. Continuous supplementation competes with the biosynthetic output of the strains themselves, drowning out their signal and undermining the basis of cooperation between the two strains.

For the continuous supplementation scheme, we provide only theoretical results. As before, we plot equilibrium values of the *ADE*↑ strain for different amounts of continuously added adenine and lysine (relative to adenine concentration) (Fig. 4, right panel). Slight, but continuous addition of lysine immediately shifts the composition of the two strains as the *ADE*↑ strain takes over the mixed culture. Compared to initial supplementation and compared at the same time point, continuous supplementation shows a drastic change in the strain composition.

### Intervention measures in the presence of an ultimate overproducer.

A common threat to a mutualistic system is the evolution of a cheater, which parasitizes the produced common good (28). A cheating strain can thrive because it benefits from public goods without having to pay the cost of contributing. However, the sharing of metabolites can be inexpensive or even free (29). If the production of the common good entails little cost, then we envision mutants that do not require mutualistic interactions but that contribute to the pool of common goods, like fictitious altruistic *ADE*↑*LYS*↑ strains. While not participating in mutualism might be lethal, taking part in a mutualistic interaction does not need to be costly if it involves the overproduction of a single metabolite. Although specific, yeast can overproduce a fluorescent protein and only suffer a 1% reduction in cost per copy (30). Moreover, reliance on external sources for essential metabolites can have a considerable cost as well. Several auxotrophic yeast strains, such as those unable to produce their own lysine or adenine, have up to a 10% reduction in growth, even with environmental supplementation (31, 32).

Typically if a strain overproduces a compound, a cost is associated with it. However, there is potentially also a cost associated with relying on interactions with other organisms. Our mathematical model can be extended to include a fictitious *ADE*↑*LYS*↑ strain that overproduces both lysine and adenine and does not require any supplementation. We assume that any cellular cost incurred will not matter unless it affects the growth rate. In the simplest case, the growth rate would be chiefly independent of the environment since the strain can satisfy its requirements.

**FIG 7:**
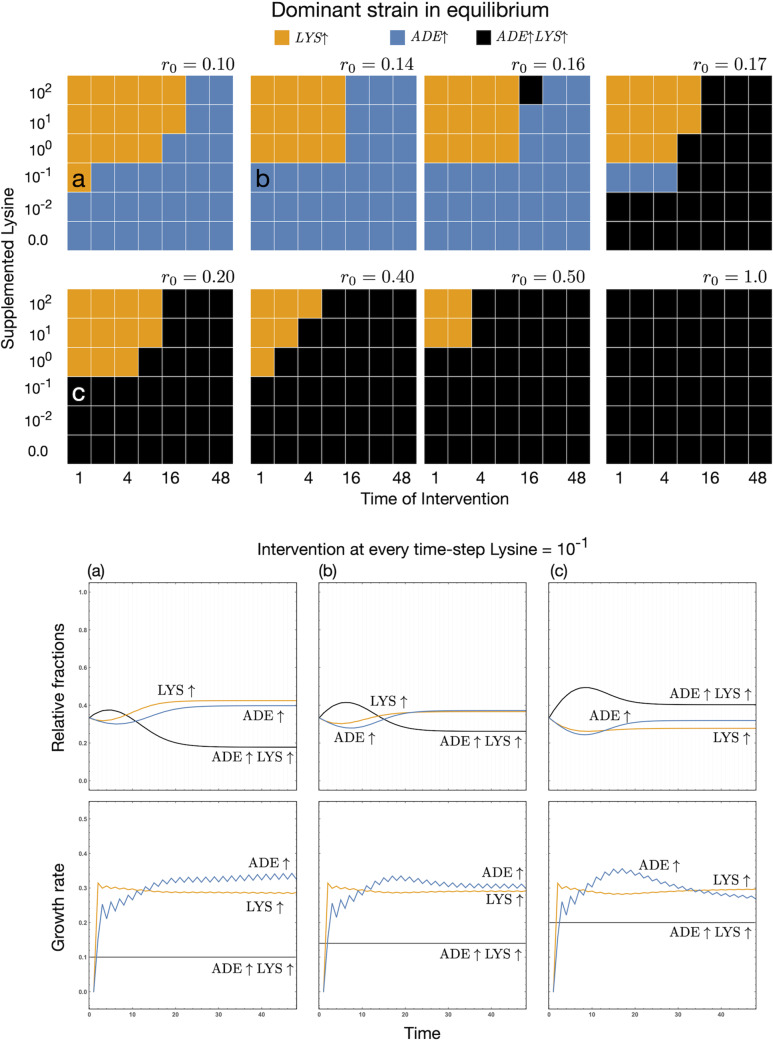
Ultimate overproducers and equilibrium dominance. By dominance we mean that one strain has a higher relative fraction in the culture. Here, we focus on intermittent supplementation which starts at a given time point and repeats after the same interval. For example, intervention at time point 2 starts supplementation at 2 and then every 2 time steps. The top grid panels show the dominant strain for a given intervention time and amount of lysine supplementation. The different grids are for the different growth rates of the *ADE*↑*LYS*↑ overproducer *r_o_* = (0.1, 0.14, 0.16, 0.17, 0.20, 0.4, 0.5, 0.7). We choose these particular growth rates to illustrate the dynamics in the dominance. Focusing on a particular grid location, one can track how *r_o_* affects the dominance. The actual dynamics for representative scenarios (a), (b), and (c) are shown in the bottom panels together with the metabolite-dependent growth rates of the *ADE*↑ and *LYS*↑ strains. The bottom rows of the grid are the dominance results when no supplementation is added. Hence, moving up the rows, we see the effect of added supplementation, whereas across each row, we see the effect of delayed intervention. To see a change from the baseline, intervention needs to be high or early if low.

Freeing itself from any environmental dependence on metabolites, our ultimate overproducer is defined as having a variable flat growth rate. Compared to growth rates extrapolated from the experimental data, we can envision several different scenarios as in Fig. 7 (top). The variable growth rates chosen for the overproducing strain reflect a range of values within those derived from the experimental data. A precise prediction for which of the three strains is dominant is derived from our updated model and closely reflects the underlying growth rates. Even with a comparatively high cost, the *ADE*↑*LYS*↑ strain can quickly become the dominant strain, despite supporting both the *ADE*↑ and *LYS*↑ strains. If we are interested in ensuring mutualism, then we must intervene, but the question is when and to what degree?

The theoretical model shows that we can supplement the culture with one of the metabolites. Since the *ADE*↑ strain has a higher growth rate, we support it by providing lysine in the culture (environment). As the amount of supplementation is reduced, it must be provided earlier to maintain the *ADE*↑ strain as the dominant strain. The more delayed the supplementation intervention, the more likely that predictions of the growth rate for unsupplemented populations hold. The same holds for scenarios in which the ultimate overproducer dominates. We need to provide a large amount of supplement early to offset the fitness benefit of the ultimate overproducer in Fig. 7. If supplementation occurs after the carrying capacity has already been reached, then it has no effect, as seen from Fig. 6.

Model dynamics including the ultimate overproducer are given by

(4) $$\begin{array}{l} {\dot{x}}_{o}=x_{o}r_{o}\left(1-\frac{x_{o}+x_{L}+x_{A}}{K}\right)\\ {\dot{x}}_{L}=x_{L}\left(r_{1}\frac{c_{A}}{c_{A}+k_{c_{A}}}\right)\left(1-\frac{x_{o}+x_{L}+x_{A}}{K}\right)\\ {\dot{x}}_{A}=x_{A}\left(r_{2}\frac{c_{L}}{c_{L}+k_{c_{L}}}\right)\left(1-\frac{x_{o}+x_{L}+x_{A}}{K}\right) \end{array}$$

where the growth rate of the overproducer is independent of the metabolite concentrations. Metabolites now also increase due to the overproducer,

(5) $$\begin{array}{l} {\dot{c}}_{A}=\beta_{1}(x_{o}+x_{A})-\frac{\gamma_{1}c_{A}}{c_{A}+k_{c_{A}}}x_{L}\\ {\dot{c}}_{L}=\beta_{2}(x_{o}+x_{L})-\frac{\gamma_{2}c_{L}}{c_{L}+k_{c_{L}}}x_{A} \end{array}$$

Thus, the growth rate of the overproducer (*r_o_*) is constant, whereas the growth rates of the *ADE*↑ and *LYS*↑ strains change over time as metabolite concentrations change. When we look for the dominant strain (the one with the highest frequency in the culture), it is not just the growth rates that need to be taken into account, but their dynamics over time in the presence of supplementation matter as shown in Fig. 7.

**FIG 8:**
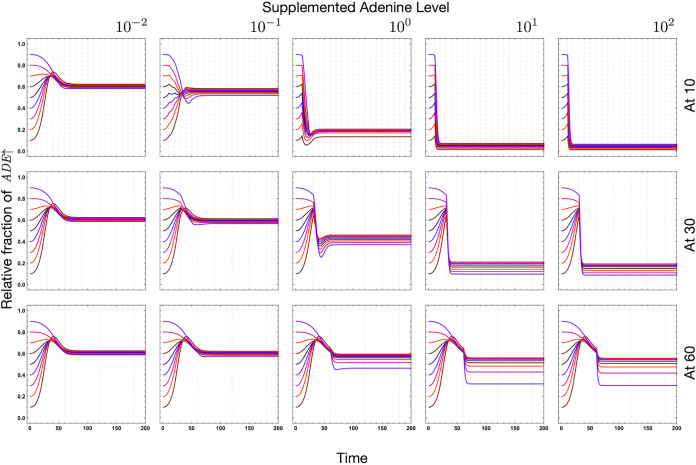
As shown in Fig. 6, the frequency and timing of intermittent supplementation change the equilibrium of the system, but the initial conditions matter as well. In this figure we highlight this dependence. Starting the supplementation regime at time point 5 and then supplementing at every 5th time point, we obtain the top row for different levels of supplementation. For the middle and bottom rows, the first dose (and subsequent doses) of supplementation occurs at 15 and 25 time points. For high supplementation, the number of cycles is immaterial. A single dose of supplementation is enough to shift the equilibrium; however, the exact timing of this dose is crucial. The time at which the first disturbance occurs affects the role of initial conditions. If supplementation begins early (at 5), then the order of initial conditions is not affected as opposed to cases in which supplementation is started later (at 15 and at 25).

In the absence of external supplementation, the system requires a specific time to equilibrate. If supplementation continues to disrupt this process by disturbing transient dynamics, then the eventual outcome when evaluated at the same time point as in other supplementation regimes can be drastically affected (see Fig. 6 and Fig. 8 for the importance of starting frequencies). In Fig. 7, compare point a (10^−1^ lysine supplementation, every time point for *r_o_* = 0.1) to no supplementation. With no intervention, the dominant strain is the *ADE*↑ strain. Intervening with a small amount of lysine (10^−1^) at later time points (in this case even time point 2 onward) does not destabilize the dominance. With higher doses, the time of intervention can be delayed (rows above point a). However, if we want to intervene to see a change in the dominance of a strain, then with minimal disturbance, we need to intervene early.

If the growth rate of the overproducer is high, it dominates the population. However, for comparable values of growth rates between the strains, the concentrations of the metabolite also matter. For *r_o_* = 0.1 under no supplementation, the growth rate of the overproducer is higher than those of the *ADE*↑ and *LYS*↑ strains. Metabolites start accumulating; the growth rates of *ADE*↑ and *LYS*↑ increase. The initial growth spurt of the overproducer ends with the dominance of the *ADE*↑ strain. In this scenario, under minimal supplementation, we can get Fig. 7 (a). We can monitor the change in dominance as we increase the growth rate of the overproducer, as shown in panels a, b, and c in Fig. 7. The dominance shifts from the *LYS*↑ strain to the *ADE*↑ strain, and eventually for a high enough *r_o_*, to the ultimate overproducer.

## DISCUSSION

Cooperation is instrumental at all levels of life and on all time scales. Although significant, the behavior of these complex interactions is challenging to study and nearly impossible to predict. Even simple interactions between a small number of cooperators when placed in responsive environments surge in complexity (33). To this end, we leverage the simplicity of a mathematical model and complement it with an engineered yeast system. Synthetic biological systems apply engineering principles to an organism to promote precise control and predictability over natural behavior. Our work examines both the effects of how initial strain ratios and changes in the environment alter the dynamics of strain concentrations and influence mutualism stability. Under stable environmental conditions, as previously shown, the initial strain ratio of the system is very stable (15). Stability under changing environmental conditions is an entirely different matter. Environmental changes have the potential to influence or disrupt mutualistic interactions (13). Here, we dissect these influences further aimed at discerning how mutualistic interactions may be maintained. Beyond interactions between biotic components, we have integrated abiotic factors, such as the amount and timing of an environmental change in our system.

The final equilibrium of the mutualistic system depends more on environmental supplementation shocks than on the starting ratio of the mutualist partners. As initial supplementation of adenine increases, favoring the *LYS*↑ strain, the final ratio shifts in both the experimental system and mathematical model (Fig. 3). Confident in the exploratory power of our mathematical model, we determined the environmental conditions that maintain the equilibrium of the system close to one observed in unsupplemented conditions. The modeled supplementation regimes are typical in microbial culturing, but they also have clear ecological parallels. The nonsupplemented culture represents a niche baseline. Initial supplementation is akin to an isolated event like the sinking of a whale carcass to the floor of the ocean, resulting in extreme point source enrichment (34). Intermittent supplementation represents seasonal or periodic change, such as temperature fluctuations or the regular introduction of a nutrient to the gut of a host animal. Understanding the effect of fluctuating resource availability is extremely important regarding invasiveness (35). A temporary reduction in competition due to nutrient excess can make mutualisms vulnerable to invasion (36). Finally, continuous supplementation is a permanent change to an environment, as in a permanent temperature change or evaporation of a large body of water. In general, we observe that as one progresses from initial to intermittent supplementation and then to continuous supplementation regimes, one of the mutualists dominates the system (Fig. 4). Tracking the size of the zone is thus a good measure of the resilience of a mutualistic system under changing environments.

The mode of intermittent supplementation, especially timing, offers some respite. These yeast strains have asymmetrical starvation tolerance and do not release their overproduced metabolite until near death (15). Intermittent supplementation could thus represent a death-induced periodic release of nutrients. It is important to note that the frequency of disturbance in complex communities can severely affect assemblages of microbes (37). We explored this by starting intermittent supplementation at different times and by changing the supplementation frequency (Fig. 5 to 7) and starting ratio of the two strains (Fig. 8). We observe that when environmental changes are minute, initial ratios matter, but if the environmental change is drastic, then the timing of the change is crucial. The longer we delay intervention, the higher the magnitude of the environmental disturbance required. Thus, if the aim is to maintain the equilibrium, then we should not intervene early, but if we wish to change the equilibrium, then we need to act in haste.

We include analysis of a hypothetical ultimate overproducer *ADE*↑*LYS*↑ strain that allows us to explore both the cost of mutualism generally and to tease apart the influence of the timing and magnitude of environmental disturbances. Although even modest costs can be rapidly selected against, it is essential to recognize that mutualism need not be expensive from a cellular point of view. Cost-free or inexpensive metabolic cross-feeding could potentially give rise to mutualism (29). Moreover, metabolic dependency, requiring the cellular uptake of public goods, can also negatively affect fitness even in the presence of supplementation (38), although the reverse can also be true (39). Thus, it can be problematic to assume static fitness for a mutualist. Depending on the fitness of *ADE*↑*LYS*↑ individuals, they can persist in a population contributing to the public goods. These individuals are not cheaters, as they support the existing mutualistic interaction but could quickly become dominant members of an ecosystem if the cost of contribution is lower than the benefits gained from not relying on other members of the ecosystem (Fig. 7). Therefore, such a strain could eventually displace obligate mutualists. Even with low fitness, the *ADE*↑*LYS*↑ strain can persist in a stable equilibrium with the other strains, in part because they are not dependent upon cycles in abiotic factors. This strain could also potentially give rise to Black Queen dynamics, which would facilitate the evolution of mutualism via gene loss in *ADE*↑ and *LYS*↑ strains (40).

It is possible to mitigate the disruptive influence of an overproducer. Intervention, early and with considerable supplementation, permits the continuation of the mutualistic interaction between *ADE*↑ and *LYS*↑ strains. In principle, targeted intervention in collapsing ecological niches that depend on mutualism could save these relationships or at least forestall their collapse. Further experimental tests along these lines will provide insight into the role of abiotic components in the resilience of mutualistic systems.

Understanding environmental enrichment (or degradation) is imperative as we face climate change. The promise of bioengineering, together with cooperation, is enormous (41). Besides offering insight into the nature of interactions resulting in community stability (42), engineered systems have implications for designing complete ecosystems affecting the biosphere (43). With continuing eutrophication of oceans, essential symbiosis may break down into parasitism (6). Use of pesticides and synthetic nitrogenous fertilizers can disrupt the natural nitrogen fixation process—mutualism between leguminous plants and rhizobacteria (4). Thus, enriched environments could pose a threat to long-evolved mutualism, which might lead to further catastrophic events (44). Since mutualisms bind organisms together to enhance their survival, they come at the cost of binding the fates of all involved species together (45). Changing the environment inadvertently or purposefully via anthropogenic activity and climate change can be costly not just to one set of species but to the ecosystem at large (20). Beyond conservation biology, mutualistic interactions also lie at the heart of translational biology, affecting applied human health and biotechnology (46).

Microbes often occur as consortia rather than as individual species. Communities of bacteria are usually the pioneers in harsh environments, from newly formed volcanic rock to hyperarid deserts (47–49). Some members of the consortia survive by consuming inorganic matter, but most of the community survives in a cross-feeding network (50). While the diversity of individuals in a consortium does not always guarantee robustness, engineered microbial consortia might prove more robust in harsh environments (41). Learning from natural, well-established communities, we can better design interactions between microbes, making them resilient to environmental disturbances, and thus useful tools in biotechnology. When developing techniques to tackle problems such as wastewater treatment, biofuel generation, or oil spill cleanups, symbiotic communities of microorganisms potentially offer an efficient pathway to the breakdown of complex substrates (51).

Understanding mutualism can, therefore, help address questions about the origin, spread, and diversification of life in inhospitable environments. While mathematical biology has long been useful in developing theories about the stability of ecosystems, synthetic microbiology will help us test these interactions. With the advent of engineered interactions, we can isolate the effects of the environment, such as poor/rich and stochastic environments. Competition between several, synthetically constructed, cross-feeding systems in complex environments may eventually lead to the evolution of successful hypercycles—as envisioned by Eigen and Schuster (21).

## MATERIALS AND METHODS

### Experimental materials. (i) Saccharomyces cerevisiae strains.

The synthetic system is composed of two metabolite-overproducing strains previously developed in the w303 background, provided by W. Shou (15). The first, WS950, is an adenine-overproducing strain referred to as *ADE*↑ throughout this manuscript. It has the genotype *MAT***a** *ste3*::*kanMX4 lys2*Δ*0 ade4*::*ADE4*(*PUR6*) ADHp-DsRed.T4. The second, WS954, is a lysine-overproducing strain called *LYS*↑ here. This strain has the genotype *MAT***a** *ste3*::*kanMX4 ade8*Δ*0 lys21*::*LYS21*(fbr) ADHp-venus-YFP where fbr stands for feedback resistance.

### (ii) Media.

Synthetic complete (SC) medium was made from dextrose, FORMEDIUM yeast nitrogen base and an appropriate synthetic complete dropout supplement (Kaiser). Amino acid-free minimal media (SC-aa) was made as above without synthetic complete dropout supplement. Yeast extract-peptone-dextrose (YPD) medium was made using chemicals from BD Diagnostics or Sigma.

### Experimental procedures. (i) Culturing.

All cultures were grown at 30°C in an orbital shaker at 200 rpm. Each experiment was performed with biological triplicates starting from a single individual colony. Experimental cultures were generated by selecting individual colonies from a streak plate and growing each colony in 5 ml SC medium for 48 h. These cultures were pelleted, washed twice with SC-aa, and resuspended in 5 ml of SC-aa. They were then grown for an additional 24 h. This allowed the culture to reach a carrying capacity of approximately 5 × 10^7^. These cultures were pelleted, washed, and resuspended as described above and were then diluted 1 in 20 with SC-aa. Thus, all experiments were batch cultures starting at approximately 1/20th of the carrying capacity. Without supplementation, experimental cultures failed to reach carrying capacity in the experimental time frames. Stock solutions of adenine or lysine were made using SC media. Supplements were added at 1 part in 500 parts. This dilution was well below detectable variation.

### (ii) Single-strain growth.

Single-strain growth tests were performed using 5-ml aliquots of the 1 in 20 dilution and supplementing to a final concentration with either 0, 0.1, 1, 10, 25, 50, 100, or 200 μg/ml of adenine for the *LYS*↑ strain or lysine for the *ADE*↑ strain. The cultures were sampled at 0 and 24 h.

### (iii) Coculturing and supplementation.

Experimental cultures were generated by mixing individual strain dilutions at volume ratios indicated in each experiment to a final volume of 5 ml. These cocultures were supplemented with either 0, 0.1, 1, 10, 25, 50, 100, or 200 μg/ml of adenine at time point 0. The cultures grew for 144 h and were sampled to determine CFU and strain ratios every 24 h (including time point 0). The 0.1, 1, 10, or 100 μg/ml adenine-supplemented cultures peaked in the number of CFU, suggesting growth saturation at 144, 120, 72, and 48 h, respectively. In each case, an equilibrium was reached before saturation. Cell viability of growing cultures was assessed using the Muse cell counter (Merck Millipore) with the Cell Count & Viability kit per the manufacturer’s instructions.

### (iv) Delayed intermittent supplementation.

Experimental cocultures were established as described above. For these experiments, a single *LYS*↑ to *ADE*↑ volume ratio, 0.65 to 0.35, was used. Cultures were supplemented at either 12 or 24 h after establishment with 0, 0.1, 1, 10, or 100 μg/ml adenine. They were then supplemented with the same amount of adenine either every 12 or 24 h (depending on the initial delay). The cultures were also sampled every 24 h for CFU and strain ratios.

### (v) CFU and strain ratios.

All experimental cultures were sampled by removing 20 μl every 24 h. These samples were serially diluted CFU determined via plate counts on YPD solid medium. Strain ratios were determined by replicate plating these YPD colonies onto solid media that permitted growth of only one strain, either SC without lysine or SC without adenine, and counting the respective colonies.

### (vi) Estimating growth parameters.

**FIG 9:**
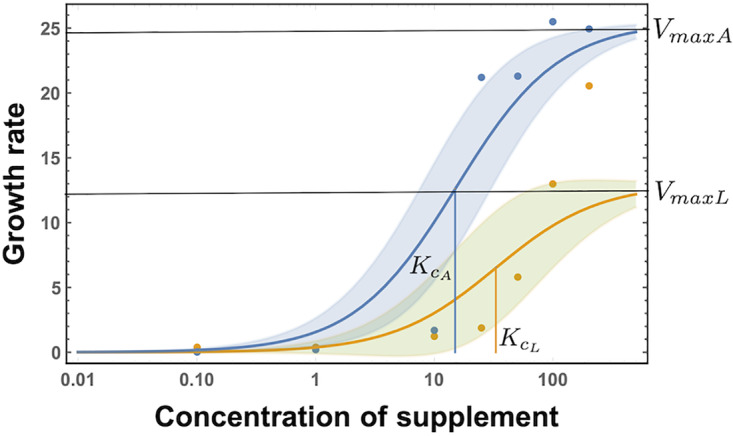
Growth curve. Experiments on growth rates for different levels of concentrations of metabolites yield the data points plotted in the figure. Each data point is the mean for biological triplicates. Thus, we understand that growth rates are dynamic in metabolite concentrations. The curves use a Michaelis-Menten representation, and we estimate the maximum growth rates *V*_max_ and the half-saturation constants *K* as in Table 1. Note that for the theoretical model we do not use any fitting. We are interested in relative amounts so as to derive a general model.

**FIG 10:**
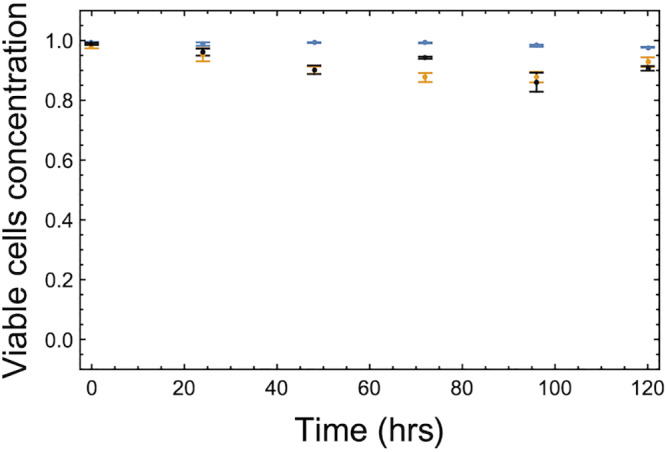
Strain viability. An assessment of strain death rates using the Muse cell counter using the Cell Count and Viability kit. Strains with either adenine or lysine starvation had very little change in viability, as a percentage of the total culture, over 120 h. Each data point is the mean for three biological replicates with error bars representing 1 standard deviation.

We now parameterize the Michaelis constants ($k_{c_{A}}$ and $k_{c_{L}}$) and the growth parameters (*r*_1_ and *r*_2_) using the single-strain growth experimental data. Single-strain growth rates for the *ADE*↑ and *LYS*↑ strains were determined on growth media supplemented with various concentrations of the appropriate required metabolite. The two strains were grown in synthetic complete media either unsupplemented or supplemented with 0.1, 1, 10, or 100 μg/ml of the corresponding metabolite. Growth rates under increasing concentrations of supplementation provided us with the supplementation-dependent growth rate function (Fig. 9). This curve was used to parametrize the functional form of growth rate [*r_i_s*/(*s* + *k_s_*] where *s* is the supplementation and *r_i_* is derived from the maximum of the function. From the raw data provided in the GitHub repository, we can estimate the maximum of the growth function of the *ADE*↑ strain to be twice as much as that of the *LYS*↑ strain. The half-saturation constant of the *ADE*↑ strain is estimated to be also twice as much as that of the *LYS*↑ strain. Thus, we use the normalized values of *r*_1_ = 1, *r*_2_ = 2, $k_{c_{L}}=1$, and $k_{c_{A}}=2$ as derived from the data and summarized in Table 1. Over the time course of the experiment, death is shown to be minimal (Fig. 10).

**TABLE 1:** Parameter values calculated from the experimental data^*a*^

Parameter	Description	Value
*r*_1_	Normalized maximum growth rate	$V_{\text{max}L}/(V_{\text{max}L})=1$
*r*_2_	Normalized maximum growth rate	$V_{\text{max}A}/(V_{\text{max}L})=25.4/13\approx 2$
$k_{c_{L}}$	Normalized half-saturation constant	$K_{c_{L}}/K_{c_{L}}=1$
$k_{c_{A}}$	Normalized half-saturation constant	$K_{c_{A}}/K_{c_{L}}=32.9/15.3\approx 2$

a From the data shown in Fig. 9, we obtain the values for maximum growth rates *V*_max_ and half-saturation constants *K* for the two strains, the *ADE*↑ and *LYS*↑ strains. For the theoretical model, we do not use these direct values, since our goal is not to quantitatively estimate the data. Using the relative quantities, we understand the differences between strain growth rates, and we present a qualitative model in the main text. The italic *L* and *A* subscripts in the variables stand for lysine and alanine, respectively.

### Availability of data and materials.

Colony counts were collated, and summary statistics were generated using R (52). An RMarkdown file containing raw data, summary statistics, all code and preliminary plots is available on the GitHub (https://github.com/tecoevo/syntheticmutualism) repository. All figures were generated using Mathematica (53) for consistency.
