# Supplementary material for: Synthetic Symbiosis under Environmental Disturbances
Source: mSystems. 2020 Jun 16;5(3):e00187-20. doi: 10.1128/mSystems.00187-20 (PMC7300358; doi:10.1128/mSystems.00187-20)
Supplement: TEXT S1 [file mSystems.00187-20-s0001.pdf]

SI: Synthetic symbiosis under environmental disturbances

Jai A. Denton[1,*] & Chaitanya S. Gokhale[2,*]

[1]Genomics & Regulatory Systems Unit, Okinawa Institute of Science & Technology, Onna-son, Japan

[2]Research Group for Theoretical Models of Eco-evolutionary Dynamics, Department of Evolutionary Theory, Max Planck Institute for Evolutionary Biology, August Thienemann Str. 2, 24306, Plön, Germany

Minimal model

Modelling simplified communities is still be mathematically challenging depending at the level of resolution that we aim for. For a quantitative match, and full parameterisation we would need to record the uptake rates of the metabolites, metabolite sharing, growth rates of the mutualists and death rates (Hart et al., 2019). The model presented in the main text is of intermediate complexity. While the included complexity results in analytical intractability, here we show the mathematical advantageous of a simpler but more abstract model.

Herein we show that while a simpler, tractable mathematical model is enough for understanding the evolution of the system, it comes at a cost of ecological, population dynamic prediction. We denote the densities of the two strains *LYS*↑ and *ADE*↑ by x_L and x_A. The densities fluctuate in a container with a carrying capacity normalised to 1. Hence we have $x_L + x_A \leq 1$, there can be empty space z. Excluding metabolites,

the dynamics of the two mutualists and empty space is given by,

$$\begin{aligned} \dot{x_L} &= x_L(r_1 x_A z - d) & \text{(S1)} \\ \dot{x_A} &= x_A(r_2 x_L z - d) & \text{(S2)} \\ \dot{z} &= -\dot{x_L} - \dot{x_A} & \text{(S3)} \end{aligned}$$

Thus the strains grow only if the other strain is present and there is empty space to grow into. Both strains die at the same constant rate d.

Relative fraction of *ADE*↑

To reduce the system even further we focus on the dynamics of the relative fraction of *ADE*↑($f = x_A/(x_L + x_A)$). In this new coordinate system, the dynamics are given by,

$$\begin{aligned} \dot{f} &= \frac{\dot{x_A} x_L - \dot{x_L} x_A}{(1-z)^2} & \text{(S4)} \\ &= -zf(1-z)(1-f)(-r_2 + f(r_1 + r_2)) & \text{(S5)} \\ \dot{z} &= (1-z)(d - f(1-f)(r_1+r_2)z(1-z)) & \text{(S6)} \end{aligned}$$

The solutions for this system lie in the eco-evolutionary space of population density $z = 1 - x - y$ and the relative fraction of *ADE*↑. The internal equilibria of the system are defined by the simultaneous solutions of the following set of equations,

$$\begin{aligned} f(r_1 + r_2) - r_2 &= 0 & \text{(S7)} \\ d - (1-f)f(r_1+r_2)(1-z)z &= 0 & \text{(S8)} \end{aligned}$$

shown in Figure S1.

For a fixed value of r_1 and r_2 we have the equilibrium value of $f = r_2/(r_1 + r_2)$.

Figure S1: **Eco-evolutionary dynamics** In the space of population density and the relative fraction of *ADE*$\uparrow$we show the eco-evolutionary dynamics under different death rates. For low death rates, the population is at carrying capacity with the equilibrium defined by the growth rates r_1 and r_2 (here $r_1 = 1$ and $r_2 = 2$ reflecting the growth rates as in the main text). The equilibria of the system are defined by the intersection of $\dot{z} = 0$ and $\dot{f} = 0$. As the death rate increases the solution for $\dot{z} = 0$ (dashed solution) shrinks and ultimately vanishes. Since $\dot{f}$ is independent of d the other solution (blue solid line) remains.

Figure S2: **Phase portrait for death** For $r_1 = 1$ we explore different values of r_2 from $(1.0, 1.2, 1.4, 1.6, 1.8, 2.0)$. Increasing from very small death rates, we observe a set of two solutions, unstable (dashed lines) and stable (full lines). With increasing death rates the stable solution reduces in the equilibrium population density up till 0.5 where the two solutions meet and annihilate each other. The only stable solution then is population extinction.

Substituting in the second equation in Eqs. (S8) we have $z = \frac{fr_1 \pm \sqrt{fr_1(fr_1 - 4d)}}{2fr_1}$. Plotting this solution set for different d provides us with the phase portrait as in Fig. S2. For large values of d the population goes extinct. However for low values, as is relavent for the experiments, we see a stable interior fixed point where both the strains can co-exist at appreciable density as seen in Fig. S2.

References

Samuel F M Hart, Hanbing Mi, Robin Green, Li Xie, Jose Mario Bello Pineda, Babak Momeni, and Wenying Shou. Uncovering and resolving challenges of quantitative modeling in a simplified community of interacting cells. *PLoS biology*, 17(2): e3000135, 2019.
