# Supplementary figures and images for: Synthetic Symbiosis under Environmental Disturbances
Source: mSystems. 2020 Jun 16;5(3):e00187-20. doi: 10.1128/mSystems.00187-20 (PMC7300358; doi:10.1128/mSystems.00187-20)

Relative fraction of ADE  $\uparrow$

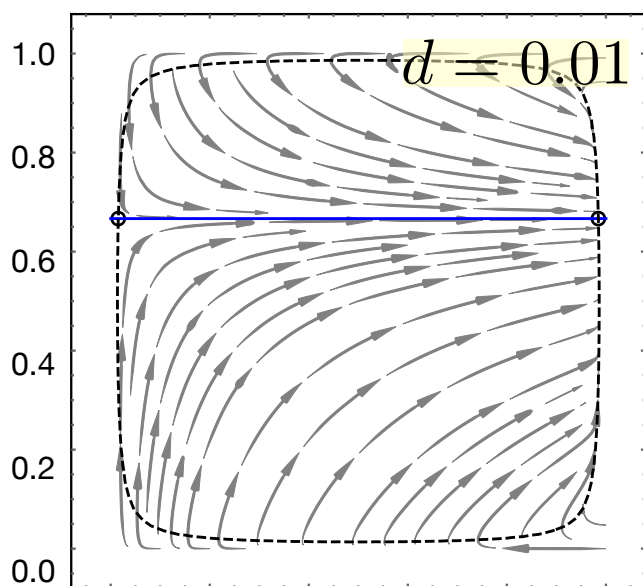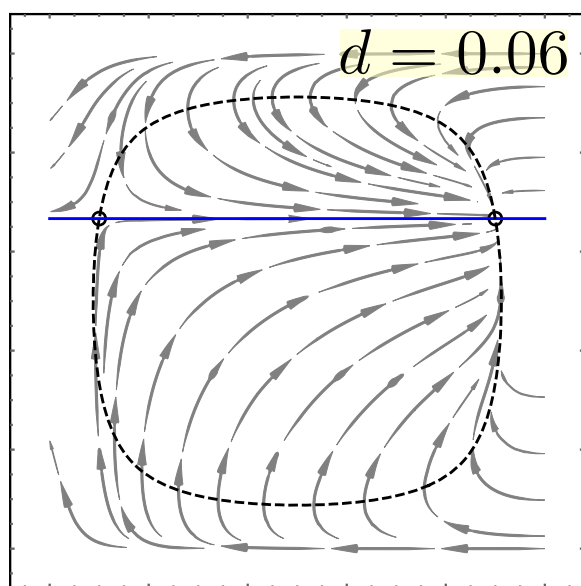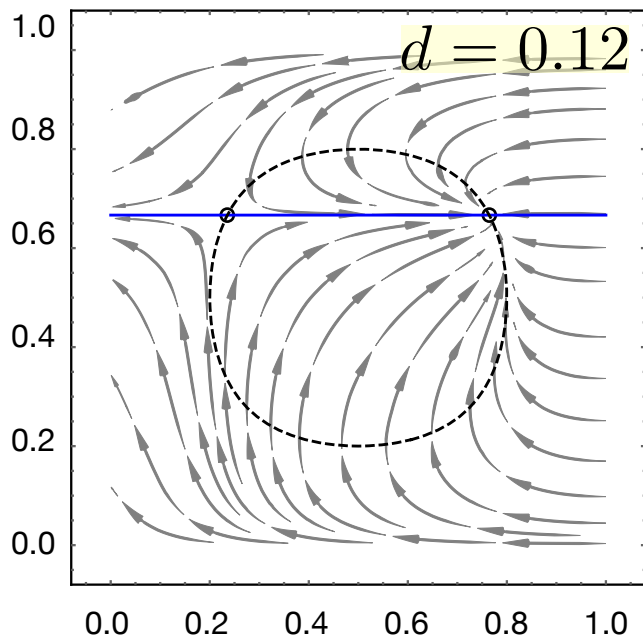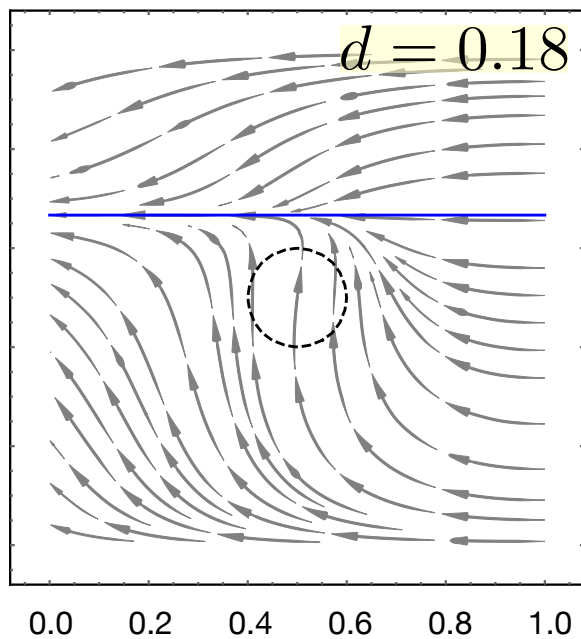

Population density ( $x_L + x_A$ )

Supplement: FIG S1 [file mSystems.00187-20-sf001.pdf]

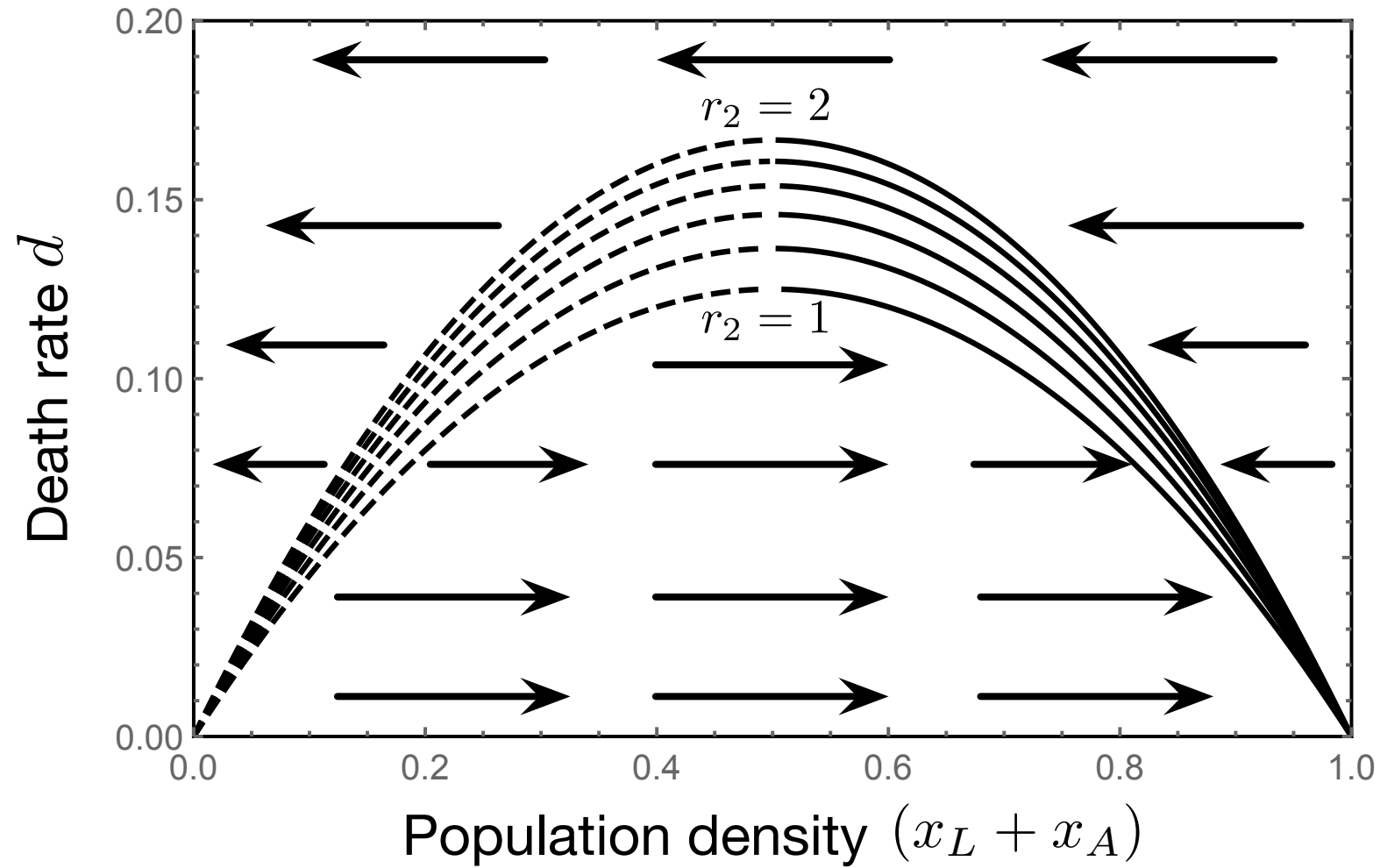

Supplement: FIG S2 [file mSystems.00187-20-sf002.pdf]
